# Isolation of intact bacteria from blood by selective cell lysis in a microfluidic porous silica monolith

**DOI:** 10.1038/s41378-019-0063-4

**Published:** 2019-06-17

**Authors:** Jung Y. Han, Michael Wiederoder, Don L. DeVoe

**Affiliations:** 1 Department of Mechanical Engineering, University of Maryland, College Park, MD 20742 USA; 2 Department of Chemical and Biomolecular Engineering, University of Maryland, College Park, MD 20742 USA; 3 Fischell Department of Bioengineering, University of Maryland, College Park, MD 20742 USA

**Keywords:** Engineering, Materials science

## Abstract

Rapid and efficient isolation of bacteria from complex biological matrices is necessary for effective pathogen identification in emerging single-cell diagnostics. Here, we demonstrate the isolation of intact and viable bacteria from whole blood through the selective lysis of blood cells during flow through a porous silica monolith. Efficient mechanical hemolysis is achieved while providing passage of intact and viable bacteria through the monoliths, allowing size-based isolation of bacteria to be performed following selective lysis. A process for synthesizing large quantities of discrete capillary-bound monolith elements and millimeter-scale monolith bricks is described, together with the seamless integration of individual monoliths into microfluidic chips. The impact of monolith morphology, geometry, and flow conditions on cell lysis is explored, and flow regimes are identified wherein robust selective blood cell lysis and intact bacteria passage are achieved for multiple gram-negative and gram-positive bacteria. The technique is shown to enable rapid sample preparation and bacteria analysis by single-cell Raman spectrometry. The selective lysis technique presents a unique sample preparation step supporting rapid and culture-free analysis of bacteria for the point of care.

## Microfluidics: Pores for thought in the treatment of bacterial infection

Scientists have developed a micro-scale device for analyzing bacteria in human blood that could enable the rapid and efficient identification of pathogens at the point-of-care, significantly improving patient outcomes. The presence of bacteria in the bloodstream can lead to serious health problems, including sepsis and infection of other tissues. Early identification of pathogens would greatly enhance the ability of clinicians to provide treatment at the earliest stages of infection, leading to improved patient care. Now, Jung Yeon Han, Michael Wiederoder, and Don DeVoe at the University of Maryland, in the United States, have developed porous silica monolith capillaries capable of selectively isolating bacteria directly from human whole blood, leaving the living bacteria intact for analysis. The millimeter-scale monoliths can be integrated into microfluidic devices, allowing rapid preparation and culture-free analysis of bacteria at the point-of-care.

## Introduction

The presence of bacteria in the blood stream can lead to serious conditions, including sepsis and infection of other tissues, and early identification of blood-borne bacteria is necessary for effective treatment selection to enhance patient outcome. The ability to rapidly identify bacteria at or near the point of care would greatly enhance the ability of clinicians to initiate optimal treatment at the earliest stages of infection. The current gold standard for bacterial characterization is based on phenotypic analysis in cell culture, requiring at least 24 h to several days between sample collection, culture and analysis in a clinical microbiology laboratory, and diagnostic answer^1–3^. While culture-based analysis is robust and inexpensive, it cannot generate timely results to guide the initial stages of treatment^4^.

Several powerful analytical methods including mass spectrometry^5^, Raman spectrometry^6^, and infrared spectrometry^7–9^ can enable culture-free identification of bacteria. However, these techniques require the isolation and purification of bacteria from the initial clinical matrix. To this end, the use of affinity capture to immobilize bacteria on magnetic beads for subsequent removal from the initial sample can be highly efficient^10–12^, but requires the introduction of reagents and instrumentation that complicate assay operation. As an alternative, size-based separation of bacteria from blood cells has been demonstrated using various microfluidic platforms employing inertial deflection^13^, inertial lift^14^, or Dean flow fractionation^15^. A central advantage associated with inertial microfluidics is that the separation may be performed in a continuous flow process, without the need for additional reagents or equipment other than a pump to deliver sample. However, inertial separations require precise control over flow rates to ensure accurate fractionation, and can only be applied to smaller bacteria with hydrodynamic radii that differ significantly from blood cells. For example, various strains of *Escherichia coli* and *Bacillus subtilis* have major axis lengths on the order of 6–8 μm^16^, similar to the diameter of human red blood cells.

An alternate strategy explored here for isolating bacteria from whole blood involves the selective lysis of blood cells under conditions that do not disrupt target bacteria, followed by a secondary size-based separation to remove bacteria from the remaining cell lysate for downstream analysis. Chemical lysis via a combination of detergent and osmotic shock can enable efficient and selective degradation of blood cells while leaving bacteria intact^17^. However, chemical lysis requires control over multiple solution flows, while chaotropic agents in the lysis buffer can adversely impact downstream assay performance. Significant dilution is also required to prevent ongoing chemical damage to target pathogens, resulting in low-bacteria concentration that can significantly complicate detection. While unwanted dilution can be reduced by repeatedly removing bulk liquid volume following sequential lysis and centrifugation steps^18^, these steps are cumbersome to implement. More fundamentally, decreased bacteria viability has been observed following chemical lysis.

In the present work, microfluidic-integrated porous silica monoliths are explored as simple flow-through elements for selective blood cell lysis and intact bacteria isolation. Monoliths are highly porous materials with an open cell morphology presenting tortuous fluid flow paths^19^. With appropriate control over monolith pore morphology, high-mechanical surface stress can be induced during cell perfusion, enabling mechanical hemolysis of blood cells while allowing intact and viable bacteria to traverse the porous flow paths. Using this approach, we show that selective passage may be achieved for bacteria in whole blood under flow conditions that yield highly efficient blood cell lysis. The technique results in smaller blood cell fragments than chemical lysis, thereby conferring a greater size difference between bacteria and lysate particles for improved downstream separation or direct detection within the lysate. Selective passage of intact and viable bacteria was achieved for gram-positive as well as gram-negative species, in spite of the thinner peptidoglycan layer associated with gram-negative bacteria that reduces mechanical robustness of the cell wall^20,21^. Finally, high throughput selective monolith lysis is combined with Raman spectrometry to demonstrate the utility of the process for culture-free analysis of bacteria in whole blood at the single-cell level.

## Results and discussion

### Porous silica monolith synthesis

**Fig. 1:**
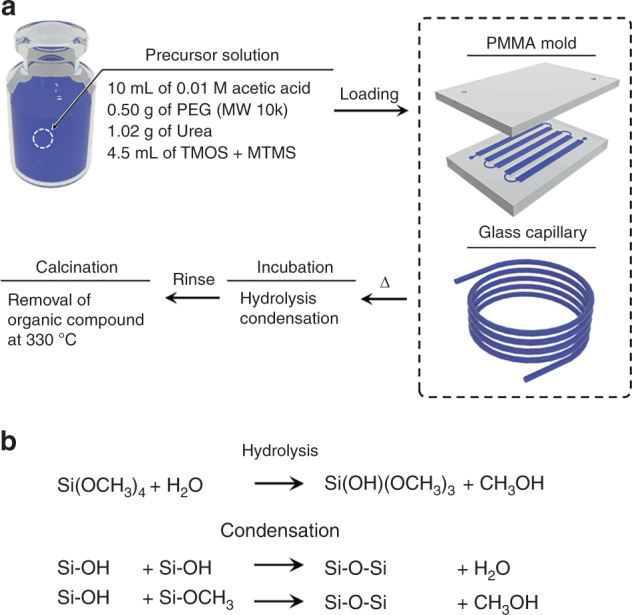
**a** Synthesis of porous silica monolith in fused silica capillary and thermoplastic mold via sol–gel chemistry. **b** Competitive reactions during the synthesis of monolith. Silanol groups present on glass capillary form covalent bonds with the monolith during this step

The silica monolith synthesis process, summarized in Fig. 1, was modified from reported studies^22–26^. Porous silica synthesis involves a competitive process of sol–gel transition and separation into a co-continuous binary phase via spinodal decomposition of a liquid mixture of alkyl silicates and porogen in acidic solution^19,26,27^. Hydrolysis and condensation of silica are the major reactions which enable the formation of silica glass from liquid alkyl silicate at relatively low temperature^28^. As the chemical reactions progress, entropic loss from the condensation of two silanol groups increases the Gibbs free energy, leading to separation into silicate-rich and solvent-rich phases. Fast hydrolysis under acidic conditions is required to uniformly hydrolyze most of the alkyl silicates, followed by a gradual increase in pH to suppress hydrolysis and boost the condensation reaction, which allows homogeneous phase separation in the mixture^19,28,29^. As described in Fig. 1, our silica monolith was prepared from a precursor solution composed of alkyl silicates, polyethylene glycol (PEG) as a porogen, urea as a source of hydroxyl ions, and acetic acid. The addition of urea was found to be important for minimizing heterogeneity of porous morphology. Unlike conventional methods for making porous silica, urea can be thermally decomposed at temperatures above 80 °C in the presence of water, leaving ammonia and carbon dioxide as products. This allows the hydrolysis and condensation reactions to be separated by increasing the solution pH during reaction, thereby obviating the need for physical infusion of basic solution which can disrupt the soft gel phase, while enabling a more uniform pH increase throughout the entire gel. A mixture of tetramethyl orthosilicate (TMOS) and methyltrimethoxysilane (MTMS) was chosen as a source of silica to overcome the intrinsic volume contraction associated with pure TMOS, in which four methoxy groups serve as crosslinking points during the condensation reaction. By using MTMS as an alkyl silicate with only three crosslinking points and one inert group, volume contraction was suppressed, allowing the porous morphology to be uniformly formed with minimal local shrinkage.

**Fig. 2:**
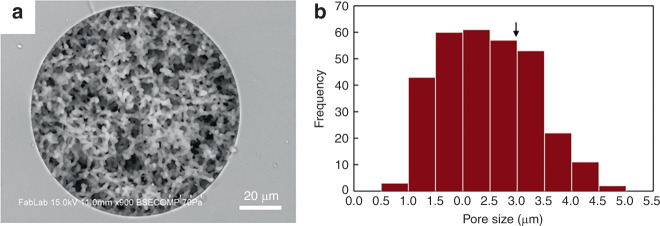
**a** SEM image of a silica monolith synthesized within a 100 μm ID fused silica capillary, revealing uniform porosity and excellent wall anchoring of the monolith. **b** Histogram of pore size. Critical diameter for RBC hemolysis (2r*) is marked with an arrow

Optimization of this process resulted in monoliths that were homogeneous and well-anchored to the silica capillary walls (Fig. 2a). The excellent attachment of porous silica to the glass capillary is attributed to covalent bonding during condensation reaction between silanol groups on the capillary wall and the growing silica gel phase within the monolith. The thickness of the final skeletal monolith structure was measured as 2.0 ± 0.3 μm, with average through-pore dimensions of 2.5 ± 0.9 μm. Permeability (*K*_*F*_) based on superficial velocity was calculated by Darcy’s law^25,30^

$$K_F = \frac{{\mu \nu _FL}}{{{\mathrm{\Delta }}P}}$$

where *μ* is viscosity of a mobile phase, *ν*_*F*_ is superficial velocity, *L* is length of the silica monolith capillary, and Δ*P* is pressure drop across the capillary. Using a high-performance liquid chromatography pump to control experimental conditions, permeability (*K*_*F*_) was found to be 2.0 × 10^−12^ m^2^. To minimize the intrinsic variation in sizes caused by hydrothermal treatment and calcination, the resulting capillary was cut into 5 cm long segments and permeability was evaluated before use.

### Porous silica monolith integration

**Fig. 3:**
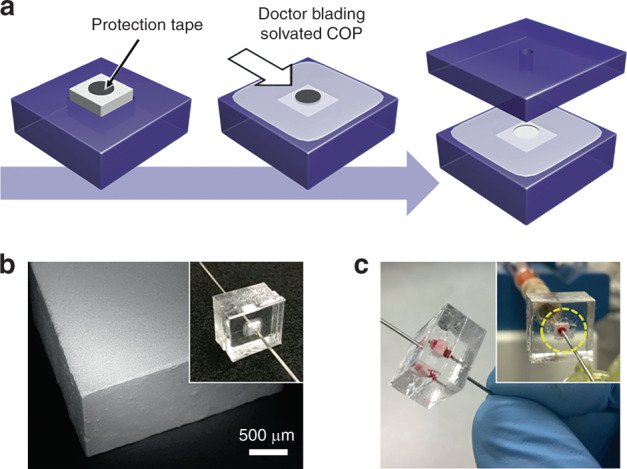
**a** Integration of a silica monolith brick into a thermoplastic chip. A circular tape is placed on a monolith inserted into a COP substrate, and solvated COP is applied to the exposed surface. After partial drying, the tape is removed, the device is enclosed by another COP substrate, and fluid ports are inserted into holes that provide a flow path through the monolith. **b** SEM image of a monolith brick cut by wafer dicing saw. **c** Image of a device during whole blood perfusion

Two complementary fabrication methods for integrating silica monoliths into microfluidic systems were developed. For low throughput operation, monolith-containing capillary segments were embedded within thermoplastic microfluidic chips, with the rigid capillary walls serving to protect the monolith during integration (Supplementary Fig. S1). To support high-throughput selective lysis, monoliths with larger cross-sectional area were integrated directly into the microfluidic devices. Centimeter-scale monolith rods prepared in a polymethylmethacrylate (PMMA) mold were cut to 2 mm length by a dicing saw, yielding small, crack-free monolith bricks (Fig. 3b). Leveraging a solvent casting technique^31^, a solution of cyclic olefin polymer (COP) dissolved in decalin was applied to the exposed surface of a monolith brick inserted into a milled cavity within a COP substrate, penetrating into the gap between the monolith and COP surface to form a permanent seal, while also supporting solvent-mediated bonding of a second COP substrate as a capping layer. Holes milled into each substrate provide a flow path through the sealed monolith. This method was found to yield excellent reliability and leak-free operation during whole blood perfusion (Fig. 3c).

### Bacterial passage and selective RBC lysis in a capillary device

To explore the efficiency of bacteria passage, we chose *Enterobacter cloacae* (gram-negative, rod shaped), one of the common cause of hospital-acquired infection^32,33^, and three gram-positive bacteria with different shapes and sizes (*Lactococcus lactis*, *Micrococcus luteus*, and *Bacillus subtilis*). Bacteria solutions were perfused through microfluidic monoliths with varying geometry and flow conditions to evaluate bacteria passage and blood cell lysis.

**Fig. 4:**
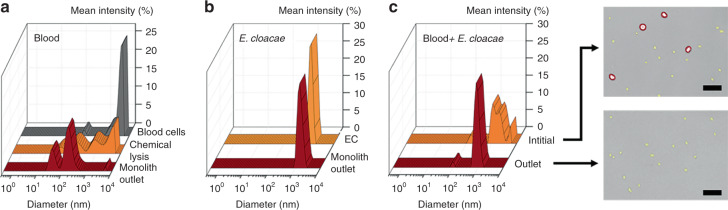
DLS measurement of **a** initial 25× diluted blood, chemically lysed blood, and blood lysed by perfusion through the monolith device, revealing a significant reduction in cell debris size for mechanical monolith lysis over chemical lysis. **b** DLS measurement of *E. cloacae* suspended in 1× PBS, and sample perfused through the monolith device, showing no change in bacteria size. **c** DLS measurement of 100× diluted blood spiked with *E. cloacae*, and sample collected from the outlet of a porous monolith. The broad peak in the inlet sample indicates a mixed population of blood cells and small bacterial cells, whereas the outlet sample showed significant reduction in large (>2 µm) cells, as confirmed in the optical images. Scale bars = 25 µm

Samples of *E. cloacae* and diluted blood were first processed through 3 mm long monolith capillary-integrated devices and analyzed by dynamic light scattering (DLS). Erythrocytes and *E. cloacae* were observed in two distinct size ranges for the unprocessed samples, with erythrocytes at 3–6 μm diameter (Fig. 4a) and bacteria at around 0.8–2 μm diameter (Fig. 4b). When analyzing 25× diluted blood, a strong peak at 3–4 μm is observed, reflecting the presence of intact red blood cells (RBCs) in the sample. After processing through the monolith, this peak is nearly eliminated. Unlike chemical lysis, which yields a broad range of particle sizes including primary peaks around 50 nm, 200 nm, 1 µm, and 4 µm, the lysate generate by the monolith exhibits only two major peaks at 50 and 200 nm. The upper peaks in the chemical lysate presumably reflect the presence of intact erythrocytes (4 µm) and larger membrane fragments (1 µm) that result from incomplete osmotic pressure-induced membrane rupture in the lysis buffer. In contrast, the mechanical lysis process nearly eliminates these larger peaks, resulting in lysate that contains smaller vesicles around 200 nm diameter and protein aggregates that appear as a 50 nm peak in both the chemical and mechanical lysates.

Perfusion of purified *E. cloacae* through the monolith yields no discernable change in the DLS peaks, indicating intact bacteria passage. Similar results were observed from tests using 100× diluted blood spiked with *E. cloacae*, as shown in Fig. 4c. Two significant peaks at around 100 nm and 1 μm match well with corresponding peaks observed with diluted blood (Fig. 4a) and purified *E. cloacae* alone (Fig. 4b).

**Fig. 5:**
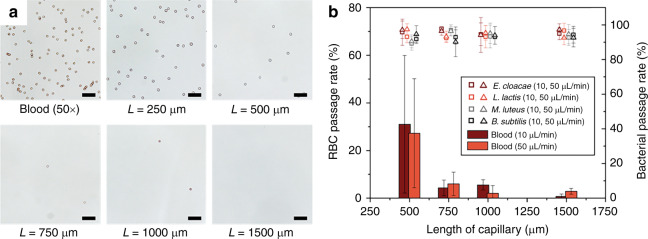
**a** Monolith length dependence of RBC hemolysis. Totally, 50× diluted blood in 1× PBS was perfused through capillary monoliths of various lengths at a flow rate of 10 μL/min. **b** Passage rate of RBC and viable bacteria at different flow rates and lengths of monolith-containing capillary. Scale bars = 50 µm. Error bars are ±1SD. Contrast of optical images was adjusted for visibility

The RBC lysis efficiency was found to be strongly affected by the porous monolith length (Fig. 5a). In particular, significant variance was observed for monolith lengths below 500 μm. Despite efforts to minimize heterogeneity of the porous network, it has been reported that silica monolith prepared from the mixture of MTMS and TMOS can exhibit up to 30% RSD for pore size^25^. The presence of larger through-pores observed in our monoliths are believed to be a significant contributor to the variance in our samples (35% RSD), and to the resulting length dependence for blood cell lysis. To minimize the impact of variable pore morphology on blood lysis efficiency, we tested different capillary monolith lengths at various flow rates. A study of RBC lysis efficiency was conducted using 25× diluted whole blood in TRIS buffer at 10 and 50 μL/min. In addition, the effects of monolith length and flow rate on the viability of bacteria were also studied in the same manner using four bacteria strains suspended in tris(hydroxymethyl)aminomethane (TRIS) buffer. To evaluate the passage rate and viability of each bacteria strains after monolith passage, cells collected from the chip exit were plated and cultured for quantification, with results summarized in Fig. 5b. The RBC lysis efficiency was found to significantly increase for monolith lengths above 1 mm. To pass through the monolith, RBCs must deform from a discoid shape to a sphero-cylinder with radial dimension equal to that of the monolith pore size. Area expansion of the cell membrane during this process significantly increases membrane tension, resulting in RBC lysis. In the case of bacteria, even when the cells are of similar dimension as the monolith pores, less cell wall expansion is required to pass through the pores due to the overall bacteria size and morphology. Furthermore, the highly cross-linked peptidoglycan layer present in both gram-positive and gram-negative bacteria enables the cells to tolerate higher stresses^20^ during monolith transport without rupturing. As a result, intact bacteria passage remained well above 90% for all monolith lengths and bacteria species tested, with no degradation in cell viability observed.

While RBCs represent the most abundant cell type in whole blood, the fate of white blood cells (WBCs) during operation of the monolith device must also be considered. In our studies, no significant peak associated with intact leukocytes was observed in the light scattering data, suggesting that WBCs are unable to pass through the monolith without being lysed. This result was confirmed by white light microscopy of the monolith effluent, with no intact WBCs detected in any of the processed samples. In another experiment, the passage of WBCs was evaluated by infusing purified WBCs centrifuged from diluted blood and stained with Calcein AM through a porous monolith. Fluorescent imaging revealed a band of fluorescence within the monolith itself, with strong signal from the entrance of the monolith to a depth of approximately 400 µm, indicating the presence of intact leukocytes within the monolith matrix. In contrast, no fluorescence was detected within the effluent, confirming the lack of intact WBCs in the processed sample.

### Monolith hemolysis

Lysis was initially expected to result from shear-induced rupture of the RBC membrane. However, the absence of lysate particles near the average pore diameter, together with a dramatic increase in pressure across the monolith when perfusing blood at lower dilution levels, suggests that mechanical hemolysis is due to isotropic tension within the cell membrane following pore occlusion. The human erythrocyte is a discoid shaped cell approximately 2–3 μm thick and 8 μm in diameter, presenting 40% greater surface area compared to a sphere of the same volume^34^. Combined with its viscoelastic cell membrane, RBCs can tolerate a high degree of deformation at a constant volume and surface area^35,36^. However, when the deformation exceeds a threshold beyond which the membrane surface area must expand to accommodate further deformation, isotropic membrane tension rapidly increases, and the cells are finally observed to lyse when their fractional area expansion reaches a value of 3%, corresponding to an isotropic membrane tension of approximately 10 mN/m^37^.

When the pore radius is small enough to confine an RBC into a sphero-cylindrical shape with a given cylindrical radius (*r*_*c*_), the volume (*Ω*_*p*_) and surface area (*Σ*_*p*_) are given by

$$\Omega _p = \pi r_c^2l + \frac{4}{3}\pi r_c^3$$

$$\Sigma _p = 2\pi r_cl + 4\pi r_c^2$$

$$= 2\frac{{\Omega _p}}{{r_c}} + \frac{{4\pi r_c^2}}{3}{.}$$

The corresponding isotropic membrane tension (*t*_0_) of an RBC traveling within a pore in an isotonic condition can be expressed as^38^

$$\frac{{t_0}}{K} + 1 = \frac{{2\Omega _p}}{{r\Sigma _p}}\left( {1 - \frac{{2t_0}}{{{\Pi}r}}} \right) + \frac{{4\pi r^2}}{{3\Sigma _p}}{,}$$

where *K* is elastic area modulus of RBC membrane (500 nN/μm)^39^, *r* is the pore radius, and *Π* is the osmotic pressure at physiological condition (1.3 × 10^6^ Pa at 25 °C)^40^.

From these expressions, it can be seen that an isolated RBC can deform to pass through a pore of radius *r* > *r*_*c*_, where *r*_*c*_ = 1.53 μm, without exceeding the critical membrane tension, but when the pore radius drops below *r*_*c*_ the system exceeds the deformation limit where further membrane expansion cannot occur without increasing tension (see Supplementary Fig. S2). When *r* is reduced below *r*_*c*_, the maximum membrane tension rapidly increases until reaching a critical value at *r* = r*, where r* = 1.48 μm, at which point the RBC isotropic membrane tension reaches the critical value of 10 mN/m and mechanical hemolysis occurs. The porous silica monoliths presented in this study possess an average pore radius of 1.24 μm, sufficiently smaller than r* to ensure high lysis efficiency even in the presence of large variance in the pore size distribution. In contrast to RBCs, the bacteria evaluated in this work do not experience lysis during passage through the monolith pores. This behavior can be explained in terms of both the cell dimensions and membrane tension limits associated with bacteria cells. Taking *E. cloacae* as an example, a minimum pore radius of 0.6 µm is required to induce deformation of the rod-shaped cells when reptating through the porous matrix. This pore size limit corresponds to less than 8% of the pore population (Fig. 2b) and is well below the mean pore radius. For the case of bacteria with larger minimum dimensions such as *M. luteus*, which may be deformed during passage through the smaller monolith pores, the peptidoglycan cell wall confers significantly higher stiffness to the bacteria^41^ compared with RBCs^42^, allowing the cells to tolerate higher levels of membrane stress without rupturing^20^.

### High-throughput bacteria passage in a thermoplastic chip

In the capillary devices, dilution of blood was required due to the limited number of pores available for RBC lysis. To extend the capacity of the monoliths to whole blood lysis, centimeter-length and 3 mm square prismatic monolith rods were prepared in a thermoplastic PMMA mold, resulting in a cross sectional area more than three orders of magnitude larger than the capillary devices. Unlike the synthesis of porous monolith within a fused silica capillary, the condensation reaction between the gel phase and the mold is prohibited due to the absence of silanol groups on the PMMA surface. Thus, the periphery of the gel was not constrained during synthesis, resulting in a surface skin layer with pores significantly smaller than the monolith bulk (Supplementary Fig. S3). The silica monolith rods were cut to a fixed length of 2 mm using a dicing saw to ensure that the bulk internal pore structure was exposed for the final monolith bricks.

**Fig. 6:**
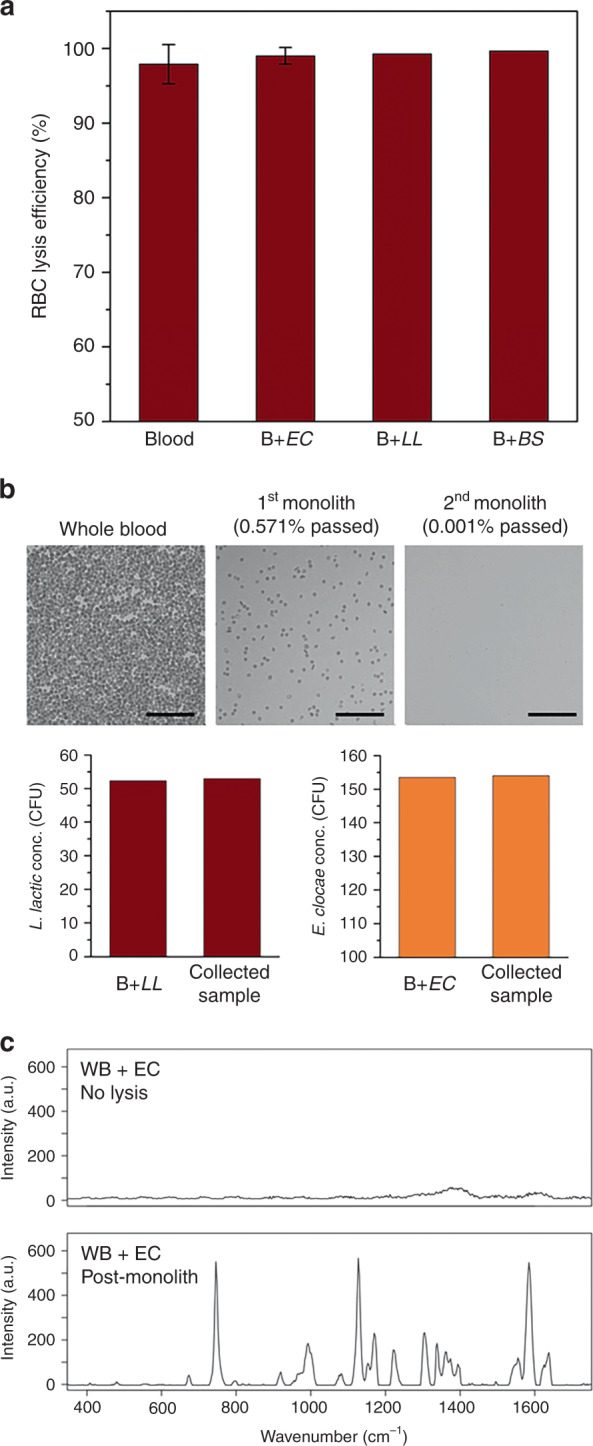
**a** RBC lysis efficiency of whole blood in high-throughput devices following perfusion at 10 μL/min (EC *E. cloacae*, LL *L. lactis*, BS *B. subtilis*. Error bars are ±SD. *N* = 3 for blood and B + EC, and *N* = 2 for B + LL, B + BS. **b** Blood cell lysis and bacterial separation following serial operation using two monoliths. Surfaces were passivated with BSA/Tween 20. Over 99.999% RBC lysis was obtained while preserving viability of *L. lactis* and *E. cloacae*. Scale bars = 100 µm. **c** Raman spectra of whole blood spiked with *E. cloacae* (upper) before and (under) after processing through porous silica monolith

Whole blood and whole blood spiked with *E. cloacae*, *L. lactis*, and *B. subtilis* were employed to evaluate blood cell lysis and bacteria passage through the integrated high throughput monolith brick devices. Whole blood samples were spiked with each bacteria suspended in 1× phosphate-buffered saline (PBS) and infused at 10 µL/min. All devices were able to process over 400 µL of whole blood before exhibiting a significant increase in back pressure, presumably due to clogging of the pores by a combination of cell debris from lysed cells and intact leukocytes trapped within the porous matrix. Collected filtrate was directly used to analyze RBC lysis efficiency, and diluted 5 times in 1× PBS for bacterial passage rate analysis to obtain 30–300 CFU per plate. In these experiments, an average RBC lysis efficiency of 99.3% was achieved, with excellent bacteria passage rates averaging 92% for all spiked strains observed (Fig. 6a). A summary comparing viable bacteria passage results for both capillary and high throughput devices is presented in Table 1.

**Table 1:** Intact bacterial passage rates for capillary monolith (1.5 mm long) and COC monolith brick (2.0 mm thick) devices

Species	Capillary device (bacteria only)	High-thoughput device (whole blood + bacteria)
*E. cloacae*	97.4 ± 3.4%	97.0 ± 5.2%
*L. lactis*	92.7 ± 5.3%	87.4 ± 0.6%
*B. subtilis*	92.7 ± 4.1%	92.5 ± 0.8%

### Serial operation and Raman detection

A single high-throughput COP device can reduce the population of intact RBCs in whole blood to less than 1% (Fig. 6a). However, due to the high density of RBCs in whole blood, this still leaves tens of thousands of intact blood cells per microliter of effluent. While extending the length of the monolith can increase lysis efficiency, the resulting fluidic back pressure renders this approach impractical. As an alternative, sequential processing of sample through multiple high throughput monolith devices was explored. In these experiments, whole blood spiked with bacteria was perfused through a monolith brick device, and the effluent was collected and introduced through a second monolith brick chip. A flow rate of 10 μL/min was used for both devices.

To inhibit bacteria adsorption during serial operation, the chip and monolith surfaces were passivated using a solution of BSA (5 wt%) and Tween 20 (0.05 wt%), with BSA serving to block primary adsorption sites and Tween 20 filling smaller voids that are sterically limiting for BSA^43,44^. While untreated devices passivated with BSA alone exhibited bacteria passage rates around 50% when perfusing low concentrations of bacteria below ca. 100 CFU/mL, the combined BSA/surfactant treatment resulted in no statistically significant loss of bacteria in the devices. Using the serial monoliths, RBC lysis efficiency was increased to 99.999%, while preserving nearly the same number of colony forming units of *E. cloacae* and *L. lactis* spiked in the initial whole blood sample (Fig. 6b).

Whole blood was next spiked with *E. cloacae* at a clinically-relevant level of 40 CFU/mL and processed through the serial monolith device. Collected effluent was deposited onto a glass slide and directly examined by confocal Raman spectrometry without any further processing. Confocal Raman spectrometry can extract phenotypic data from single purified bacterial cells^45–47^, with spectral fingerprinting capable of providing specific and accurate pathogen identification at multiple taxonomic levels^45^. As expected, the Raman spectra for the unprocessed samples were highly convoluted due to the confounding influence of intact blood cells, resulting in a uniform background signal that prevented the detection of bacteria within the complex matrix. Following background subtraction, the resulting spectra revealed no significant peaks when attempting to focus on bacteria within the deposited sample. In contrast, individual bacteria from the monolith-processed sample were readily selected by the confocal Raman optics. While the Raman background generated from the selectively-lysed sample was similar to the unprocessed whole blood sample, spectra generated from the bacteria were highly differentiated with minimal background signal. The resulting spectra revealing distinctive peaks associated with gram-negative bacteria as presented in Fig. 6c, including amide I near 1660 cm^−1^, amide II near 1575 cm^−1^, amide III near 1255 cm^−1^, major C–H stretching and CH_2_ deformation vibration at 1300–1500 cm^−1^, and adenine near 785 cm^−1^ ^48–50^.

In the present work, single-cell Raman analysis was performed by manually scanning the optical probe over the sample surface to locate target bacteria within monolith-processed sample deposited onto a glass slide. However, because this scanning process is time consuming at low bacteria concentrations, future clinical application of the selective lysis technology will benefit from a downstream step to isolate small numbers of intact bacteria at predefined locations, enabling confocal Raman microscopy to be performed without the need for scanning the optical probe over a large sample region. Finally, we note that while existing confocal Raman microscopy tools are largely confined to research environments, emerging miniature and handheld systems based on compact lens packages^51^ and confocal fiber optic probes^52^ have the potential to open the door to rapid and portable bacteria analysis directly at the point of care.

## Materials and methods

### Materials

TMOS (≥99%), ethanol (≥99.5%), methanol (≥99.8%), isopropanol (≥98%), acetic acid (ACS reagent, ≥99.7%), PEG (10 kDa average mw), urea, TRIS, PBS (tablet), bovine serum albumin (BSA, powder), and Tween 20 were purchased from Sigma-Aldrich (St. Louis, MO, USA). MTMS (97%), sodium hydroxide (10 N, aqueous), and decalin (cis + trans, 98%) were obtained from Alfa Aesar (Haverhill, MA, USA). *M. luteus*, *B. subtilis*, and *L. lactis* were obtained from the United States Department of Agriculture (USDA) Agricultural Research Service (ARS). *E. cloacae*, tryptic soy broth (TSB), lysogeny broth (LB), nutrient broth (NB), and yeast extract were purchased from Carolina Biological (Burlington, NC, USA). Fused silica capillary tubing, 100 μm ID and 360 μm OD, was purchased from Molex Connector (Lisle, IL, USA). Upchurch fittings were purchased from IDEX Health & Science LLC. (Oak Harbor, WA, USA). Iridium heat shrink tubing (500 μm initial ID, 250 μm final ID) was obtained from Cobalt Polymers (Cloverdale, CA, USA). Thermoplastic 1020R COP pellets were purchased from Zeon Chemicals (Louisville, KY, USA).

### Preparation of capillary and PMMA molds

Fused silica capillary was cut into 1 m long segments, then infused with 1 M sodium hydroxide solution. Each capillary segment was sealed with upchurch fittings, then incubated for 3 h in 40 °C oven. After incubation, the segment was thoroughly rinsed with deionized water and methanol, and dried in vacuum oven for 6 h at 50 °C. A mold for large silica monolith brick synthesis was prepared by micromachining PMMA plates using a computer numerical control (CNC) milling machine (MDX-650A; Roland, Lake Forest, CA, USA). PMMA substrates were cleaned by sonication in deionized water, and dried overnight in a vacuum oven. Metastable thermal bonding was achieved by exposing both PMMA substrates to UV/ozone in a surface decontamination system (PSD-UV, Novascan, Ames, IA, USA) for 1.5 min and pressing the substrates together under 400 psi pressure at 75 °C for 15 min.

### Silica monolith preparation

The sol–gel precursor mixture was prepared by adding 0.5 g of PEG and 1.015 g of urea into 10 mL acetic acid (0.01 M), and stirring vigorously in an ice bath. Once the components were fully dissolved, TMOS and MTMS 85:15 v/v were mixed and 4.5 mL of the mixture was added to the acetic acid solution and stirred for 40 min in an ice bath. The mixed precursor solution was then infused through a 0.22 μm PVDF syringe filter (Millipore, Billerica, MA, USA) into either a silica capillary segment or into a PMMA mold through the same syringe filter. The infused capillary and mold were tightly sealed with Upchurch capillary fittings to prevent solvent evaporation, and thermally treated at 40 °C for 12 h, then gradually heated to 80 °C over a 6 h period, followed by 15 h incubation at 80 °C to trigger urea decomposition. After thermal treatment, the silica capillary or PMMA mold was allowed to slowly cool to room temperature by natural convection.

To finish preparation of the capillary monoliths, the capillary mold was cut into approximately 12 cm long segments after discarding about 10 cm from each end, and submerged into a large methanol bath for 72 h, followed by submersion in a deionized water bath for 24 h while replacing each solvent every 6 h to remove unreacted reagents. For monoliths synthesized in a PMMA mold, the mold was opened by gently pushing a razor blade into bonding interface, the silica gel rods were collected and rinsed by submersion into a methanol bath for 24 h and deionized water bath for 24 h, with fresh solvents replaced every 6 h.

Rinsed capillaries and silica gel rods were dried at 60 °C in an oven for 12 h under atmospheric pressure, then moved into a high-temperature furnace. Calcination was completed by heating at 330 °C for 25 h, removing organic components and leaving porous silica structures. For the capillary segments, an additional 2 cm length from each end was discarded. The remaining middle portion was cut to 5 cm length for quality evaluation, then cut to the desired lengths ranging from 500 to 1500 μm using a wafer dicing saw (model 1006A; Micro Automation) equipped with a diamond blade. Porous silica rods were similarly cut into 2 mm long bricks using the dicing saw.

### Microfluidic device fabrication

Porous silica monolith capillary segments were integrated into COP thermoplastic chips using the process described in Supplementary Fig. S1. Upper- and lower-COP substrates were patterned with a CNC milling machine, using 150 and 380 μm diameter end mills to form a short region in the middle and large mating channel features on both substrates, respectively. The channels in each substrate were machined to a depth of 250 μm, except for short regions with a depth of only 75 μm. This shallow region serves as a capillary clamp designed to deform during chip bonding, such that reflow of polymer in the clamp region fully seals the flow path around a capillary placed in the channel. After machining the chip substrates, each layer was cleaned by sonication in methanol, acetone, and deionized water, then dried overnight in a vacuum oven. Both substrates were next exposed for 1.5 min to a decalin/ethanol (33/67 vol%) solution, which serves to enable solvent bonding of the COP layers and to soften the capillary clamp structures to support polymer reflow during the bonding process (Supplementary fig. S1). After solvent exposure, a capillary segment was manually inserted into the lower substrate channel with the center of the capillary approximately aligned to the capillary clamp structure. The upper substrate was finally placed onto the lower substrate, with the capillary serving to self-align both layers, and the assembly was placed in a hot press to apply a pressure of 500 psi for 5 min at 35 °C.

For selected experiments, capillary segments were also assembled with empty capillaries using heat shrink tubing, enabling simpler fluidic interfacing and quicker device fabrication. A porous silica monolith capillary element was placed into a heat shrink tubing segment, with a long inlet capillary abutting the monolith element on end and a short-outlet capillary on the other end. The assembly was then placed in an oven at 120 °C for 5 min to activate the heat shrink tubing and permanently seal the flow path (Supplementary Fig. S4).

For high-throughput device fabrication, porous silica monolith bricks were integrated into COP thermoplastic chips by the process shown in Fig. 3. Prior to milling, COP pellets were formed into 4 mm and 2 mm thick plaques using a hot press. Using a CNC milling machine, a 2 mm diameter and 3 mm deep hole was first formed in a 4 mm thick lower substrate, together with a 3 mm square and 2 mm deep socket for the monolith brick, resulting in a square socket for monolith insertion and a 1 mm deep, 2 mm diameter indentation at the bottom of the socket. An additional 3.2 mm wide and 100 μm deep slot was milled around the perimeter of the socket. This slot served as a receptacle for solvated COP to improve monolith sealing during the final bonding step. Next, a 2 mm diameter and 1 mm deep hole was milled in a 2 mm thick upper COP substrate, and needle ports for external fluidic interfacing were formed on both substrates using a 650 μm drill bit to connect inlet and outlet needles to the device. Each COP substrate was sonicated in methanol, acetone, and deionized water, and dried overnight in a vacuum oven. A 2 mm diameter circular section of pressure-sensitive wafer dicing tape (blue tape) was patterned using a 2 mm diameter PDMS punch and adhered to the center of a porous silica brick to protect the fluidic path during monolith integration. In this process, the lower-COP substrate was exposed to a decalin/ethanol (33/67 vol%) solution for 2 min, and the silica monolith brick was manually inserted into the monolith socket. After insertion, a solvated COP solution, prepared by slowly dissolving COP pellets in decalin to a concentration of 30 wt%, was applied to the lower substrate with inserted silica brick using a doctor blade. The lower substrate was dried for 2 h at room temperature to evaporate decalin and to solidify the solvated COP, after which the protection tape was carefully removed from the monolith. Next, the lower and upper substrates were exposed to the same decalin/ethanol solution for 45 s and 2 min, respectively, and bonded together in a hot press at 300 psi for 5 min at 35 °C. Bonded devices were dried overnight in a 60 °C oven to remove excess solvent.

### Selective blood cell lysis and bacteria passage

For blood lysis tests, whole blood was collected in a 6 mL vacuum container with K2 EDTA sprayed on the interior, and diluted with 1× PBS solution to specified levels prior to use. Four bacteria were selected to investigate selective passage through the silica monoliths, namely *E. cloacae* (gram negative, 0.3–0.6 μm diameter and 1–2 μm length), *B. subtilis* (gram positive, 0.25–1.0 μm diameter and 4–10 μm length), *L. lactis* (gram positive, 0.5–1.5 μm diameter coccus or ovoid), and *M. luteus* (gram positive, 0.5–3.5 μm diameter coccus). Each bacteria strain was obtained as lyophilized powder, and grown in TSB, LB, NB media for 24 h at 25–30 °C with gentle agitation. Resulting bacteria suspensions were pelletized by centrifugation, and re-suspended with autoclaved 1× PBS solution after discarding supernatant. To quantify passage of viable bacteria through the silica monolith devices, for each experiment both the initial sample and collected monolith effluent were plated, with the sample concentration adjusted to yield 30–300 CFU per plate after culture. This approach ensured an accurate determination of viable bacteria counts before and after each experiment without relying on an indirect quantification method such as live/dead staining.

### Raman confocal microscopy

Raman spectra were measured using a Raman confocal microscope (LabRam ARAMIS, Horiba Jobin Yvon, Edison, NJ, USA) equipped with a 532 nm laser source. Each sample was prepared by depositing a 10 μL aliquot onto a glass slide, then allowing it to dry in air under a ventilation hood for 2 h. All spectra were acquired over a range of 330–3034 cm^−1^, with a spectral resolution of 2.64 cm^−1^. Detector temperature was maintained between −65 and −70 °C. The laser source was focused on target cells manually, and individual measurements were taken for 10 s then averaged over six successive acquisitions. To enhance the identification of spectral features, the background for each raw Raman signal was determined by applying a peak clipping technique based on the sensitive nonlinear iterative peak (SNIP) algorithm and implemented in the R statistical computing language. The final spectra were generated by subtracting the SNIP-defined background from the raw spectrum, as demonstrated in Supplementary Fig. S5.

## Conclusion

Porous silica monoliths enable highly effective mechanical blood cell lysis, while efficiently passing intact gram-positive and gram-negative bacteria without measurable change to their viability. The integration of millimeter-scale monoliths into microfluidic flow cells supports high throughput processing of whole blood without dilution, with serial passage through multiple monoliths lysing all but 0.001% of RBCs in the initial sample and allowing nearly 100% of bacteria to be recovered for downstream analysis. In particular, selective lysis provides a robust and effective approach to sample preparation prior to Raman analysis of individual target organisms. The flow-through technique is also amenable to future coupling with nanofluidic size-based filtration methods, further simplifying spectroscopic identification by isolating and concentrating the bacteria within defined locations of the filtration device, eliminating the need to scan over a large surface to locate individual cells. In particular, this functionality would support bacteria analysis at the earliest stages of infection, where blood concentrations may be in the range of several CFU/mL, enabling effective capture and detection of single bacteria processed within the 400 µL volumetric limit of the current monolith devices. For applications where it may be desirable to process larger blood volumes, multiple monolith devices may be operated in parallel with little impact on system size due to the small size scale of the monolith devices. Finally, we note that the monoliths themselves are mechanically robust and durable following integration into the thermoplastic microfluidic cells, and the monolith brick synthesis technique is highly scalable using batch processing methods, making the resulting devices cost effective to fabricate in large quantities as disposable sample preparation elements.

## Supplementary information

Supplemental Material
