# Supplementary material for: Isolation of intact bacteria from blood by selective cell lysis in a microfluidic porous silica monolith
Source: Microsyst Nanoeng. 2019 Jun 17;5:30. doi: 10.1038/s41378-019-0063-4 (PMC6572753; doi:10.1038/s41378-019-0063-4)
Supplement: Supplementary file 1 — Supplemental Material [file 41378_2019_63_MOESM1_ESM.docx]

**Isolation of intact bacteria from blood by selective cell lysis in a microfluidic porous silica monolith**

**Jung Y. Han^1,3^, Michael Wiederoder^2^, and Don L. DeVoe^1,2,3*^**

*Correspondence: Don L. DeVoe (ddev@umd.edu)*

*^1^ Department of Chemical and Biomolecular Engineering, University of Maryland, College Park, MD 20742*

*^2^ Fischell Department of Bioengineering, University of Maryland, College Park, MD 20742*

*^3^ Department of Mechanical Engineering, University of Maryland, College Park, MD 20742*

# Supplementary Information


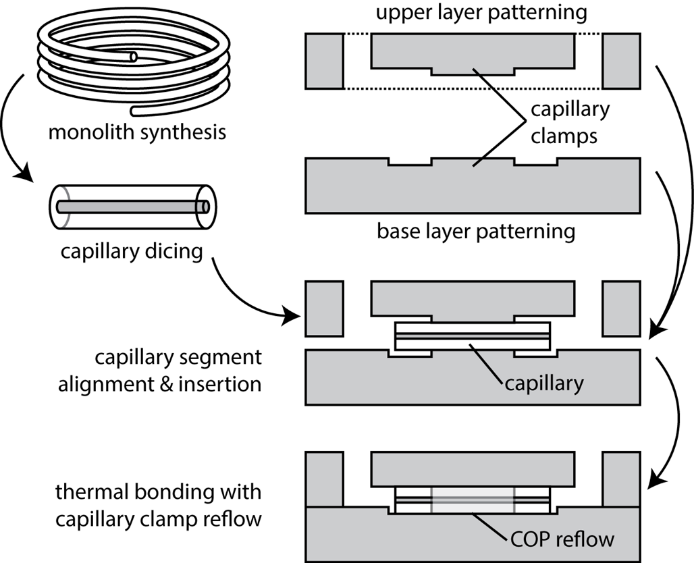


Fig. S1. Fabrication process for a capillary-integrated monolith chip. Following monolith synthesis and capillary dicing to form short monolith elements, the capillary segments are inserted into a thermoplastic COP chip containing a clamp zone that reflows during solvent bonding to fluidically seal the capillary.


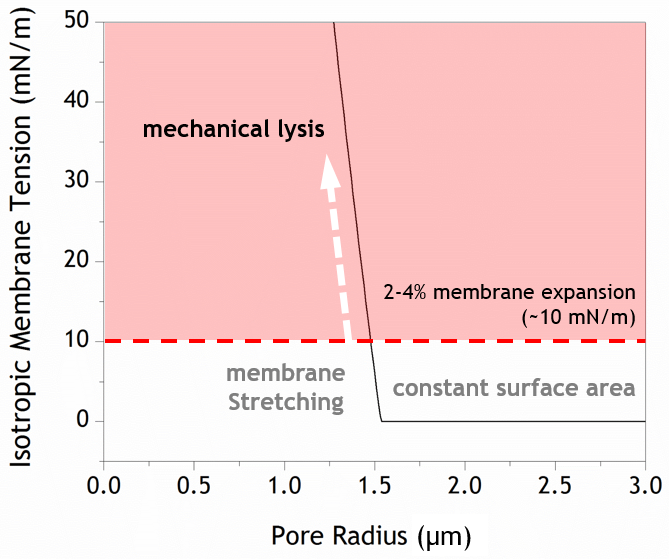


Fig. S2. Isotropic membrane tension as a function of pore radius. Membrane tension is negligible when the pore radius is within the cell’s deformable range, but rapidly increases beyond 10 mN/m, corresponding a membrane expansion of between 2-4%, when the pore radius falls below a critical radius estimated as r_c_, = 1.53 µm.


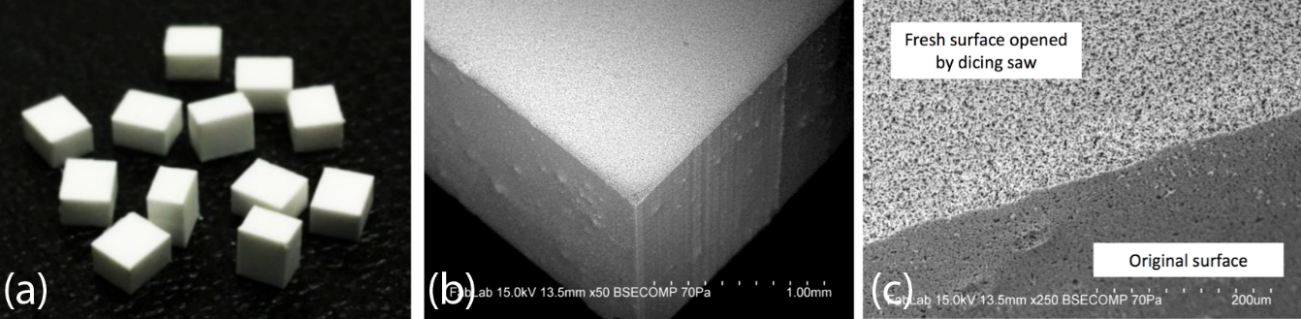


Fig. S3. (a) Monolith bricks patterned by wafer dicing. (b) Magnified view of a brick corner. The upper surface reveals the diced surface, while the two side surfaces were formed by contact with the mold. (c) SEM image revealing the desired porosity of the diced monolith surface, in comparison to the closed pores observed on the surfaces in contact with the mold during sol-gel synthesis.


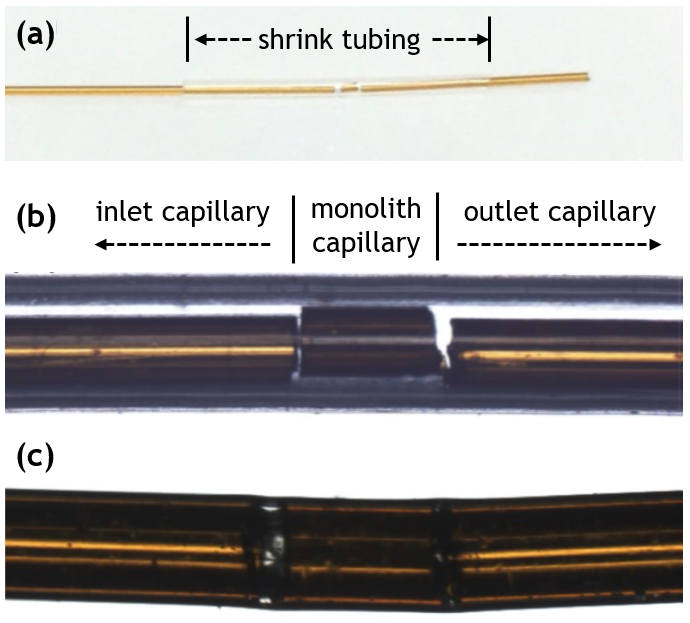


Fig. S4. Assembly of a capillary monolith device using heat shrink tubing. (a) A porous silica monolith element placed into a heat shrink tubing, with a long inlet capillary abutting the monolith element on end and a short outlet capillary on the other end. (b) Magnified view of the assembly before heat activation. (c) The final device after heating at 120 °C for 5 min.


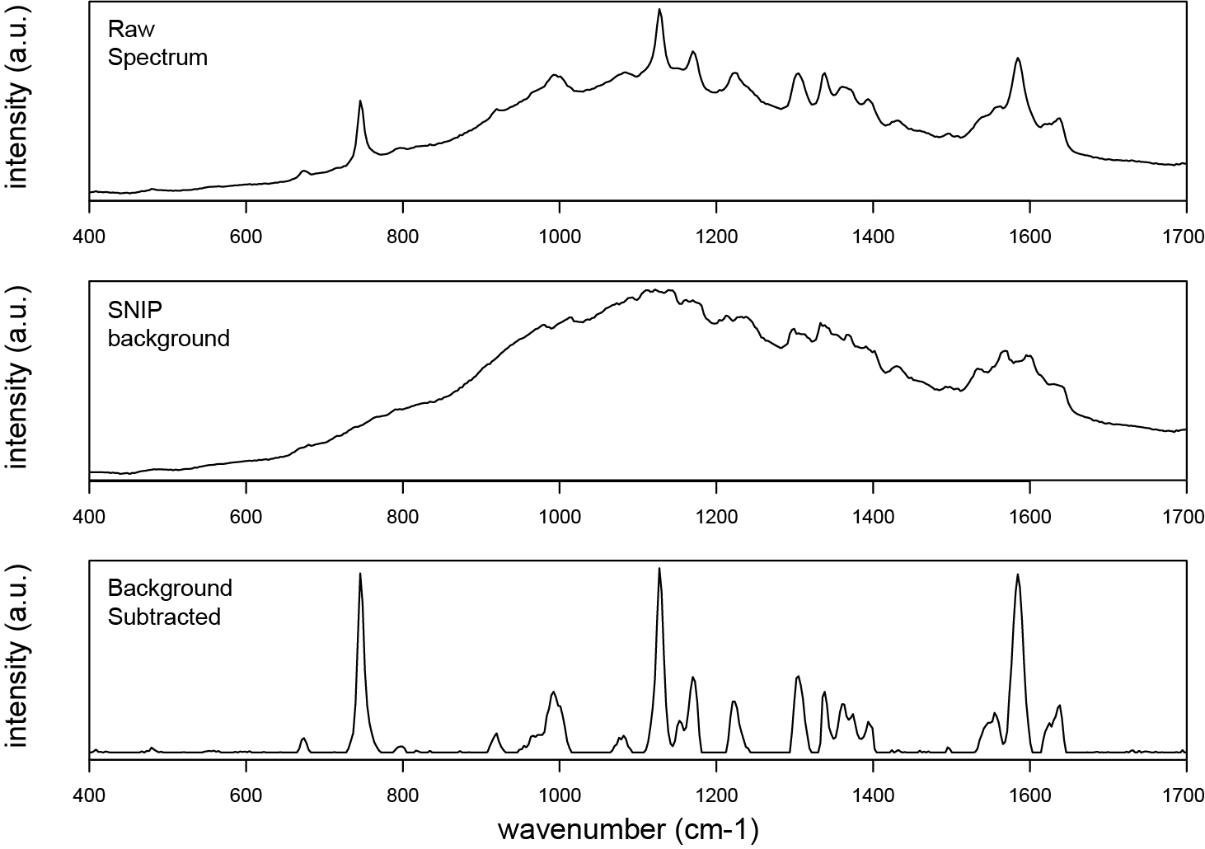


Fig. S5. Background subtraction using the SNIP algorithm. (top) Raw spectral data of a sample after processing through a silica monolith. (middle) Background spectrum estimated by SNIP algorithm, and (bottom) final background-subtracted spectrum.
